# Dynamic contrast-enhanced MRI of synovitis in knee osteoarthritis: repeatability, discrimination and sensitivity to change in a prospective experimental study

**DOI:** 10.1007/s00330-021-07698-z

**Published:** 2021-02-16

**Authors:** James W. MacKay, Faezeh Sanaei Nezhad, Tamam Rifai, Joshua D. Kaggie, Josephine H. Naish, Caleb Roberts, Martin J. Graves, John C. Waterton, Robert L. Janiczek, Alexandra R. Roberts, Andrew McCaskie, Fiona J. Gilbert, Geoff J. M. Parker

**Affiliations:** 1 Department of Radiology, University of Cambridge, Cambridge, UK; 2 Norwich Medical School, University of East Anglia, Norwich Research Park, Norwich, NR4 7UY UK; 3 Bioxydyn Limited, Manchester, UK; 4 Department of Radiology, Norfolk & Norwich University Hospital, Norwich, UK; 5 Centre for Imaging Sciences, Division of Informatics Imaging & Data Sciences, School of Health Sciences, Faculty of Biology Medicine & Health, University of Manchester, Manchester Academic Health Sciences Centre, Manchester, UK; 6 Clinical Imaging, GlaxoSmithKline, London, UK; 7 Antaros Medical, Uppsala, Sweden; 8 Division of Trauma & Orthopaedics, Department of Surgery, University of Cambridge, Cambridge, UK; 9 Centre for Medical Image Computing, Department of Medical Physics and Biomedical Engineering, University College London, London, UK

**Keywords:** Magnetic resonance imaging, Osteoarthritis, Synovitis, Perfusion

## Abstract

**Objectives:**

Evaluate test-retest repeatability, ability to discriminate between osteoarthritic and healthy participants, and sensitivity to change over 6 months, of dynamic contrast-enhanced magnetic resonance imaging (DCE-MRI) biomarkers in knee OA.

**Methods:**

Fourteen individuals aged 40–60 with mild-moderate knee OA and 6 age-matched healthy volunteers (HV) underwent DCE-MRI at 3 T at baseline, 1 month and 6 months. Voxelwise pharmacokinetic modelling of dynamic data was used to calculate DCE-MRI biomarkers including *K*^trans^ and IAUC_60_. Median DCE-MRI biomarker values were extracted for each participant at each study visit. Synovial segmentation was performed using both manual and semiautomatic methods with calculation of an additional biomarker, the volume of enhancing pannus (VEP). Test-retest repeatability was assessed using intraclass correlation coefficients (ICC). Smallest detectable differences (SDDs) were calculated from test-retest data. Discrimination between OA and HV was assessed via calculation of between-group standardised mean differences (SMD). Responsiveness was assessed via the number of OA participants with changes greater than the SDD at 6 months.

**Results:**

*K*^trans^ demonstrated the best test-retest repeatability (*K*^trans^/IAUC_60_/VEP ICCs 0.90/0.84/0.40, SDDs as % of OA mean 33/71/76%), discrimination between OA and HV (SMDs 0.94/0.54/0.50) and responsiveness (5/1/1 out of 12 OA participants with 6-month change > SDD) when compared to IAUC_60_ and VEP. Biomarkers derived from semiautomatic segmentation outperformed those derived from manual segmentation across all domains.

**Conclusions:**

*K*^trans^ demonstrated the best repeatability, discrimination and sensitivity to change suggesting that it is the optimal DCE-MRI biomarker for use in experimental medicine studies.

**Key Points:**

*• Dynamic contrast-enhanced MRI (DCE-MRI) provides quantitative measures of synovitis in knee osteoarthritis which may permit early assessment of efficacy in experimental medicine studies.*

*• This prospective observational study compared DCE-MRI biomarkers across domains relevant to experimental medicine: test-retest repeatability, discriminative validity and sensitivity to change.*

• *The DCE-MRI biomarker K*^*trans*^ *demonstrated the best performance across all three domains, suggesting that it is the optimal biomarker for use in future interventional studies.*

**Supplementary Information:**

The online version contains supplementary material available at 10.1007/s00330-021-07698-z.

## Introduction

Inflammation of the synovial membrane (synovitis) is common in OA, with MRI-detected synovitis occurring in up to 90% of OA knees [1, 2]. It can be detected, both histologically and on imaging, from the early stages of the disease [3]. Strong cross-sectional associations exist between the presence of synovitis and the severity of knee pain [2]. Longitudinal associations have been demonstrated between the presence and severity of synovitis and both symptomatic and structural OA progression [4–6]. There is therefore a strong rationale for therapeutic targeting of synovitis to provide disease modification, particularly in patients with mild to moderate disease where disease-modifying and regenerative approaches are targeted [7].

Dynamic contrast-enhanced magnetic resonance imaging (DCE-MRI) aims to characterise the uptake and washout of gadolinium-based contrast agents (GBCA) in tissues of interest, providing biomarkers of tissue perfusion, capillary permeability and blood and interstitial volume. These parameters are known to change in the synovium in OA [8]. DCE-MRI has been used to assess synovitis in early-phase clinical trials of rheumatoid arthritis and has demonstrated superiority over semiquantitative assessments in this setting [9, 10]. The promise of DCE-MRI in OA has been illustrated by several studies demonstrating changes in DCE-MRI biomarkers following intra-articular corticosteroid treatment with improved responsiveness compared to alternative semiquantitative and qualitative assessments of synovitis [11, 12].

DCE-MRI biomarkers are of particular interest in early-phase experimental medicine studies which aim to establish early proof-of-concept evidence of efficacy of novel treatments, streamline the treatment development process and reduce late-stage failure rates. They could improve outcome assessment in studies of synovitis-targeted therapies by quantifying response to treatment and are likely to be more robust than relying on qualitative or semiquantitative assessment. There may also be a role in selecting which patients are suitable for entry into studies of synovitis-targeted treatments.

However, to increase confidence in the utility of DCE-MRI biomarkers in these settings, technical and clinical validation is essential [13]. This includes an assessment of test-retest repeatability, ability to discriminate between knee OA and normal ageing and expected changes over relevant follow-up periods.

Therefore, the purpose of this study was to evaluate the test-retest repeatability, ability to discriminate between osteoarthritic and healthy participants and sensitivity to change over 6 months, of DCE-MRI biomarkers in knee OA.

## Methods

### Participants

The study was approved by the local research ethics committee, and written informed consent was given by all participants. This was a single-centre, prospective experimental feasibility study where DCE-MRI was the intervention.

Participants with mild-moderate knee osteoarthritis (OA) were recruited from specialist orthopedic knee clinics at a university teaching hospital. Healthy volunteers (HV) approximately matched for age were recruited via paper and electronic advertisement materials and from a register of healthy individuals who had agreed to be contacted about research studies. Inclusion criteria for OA participants were (i) age 40–60 years, (ii) body mass index (BMI) of ≤ 35 kg/m^2^, (iii) clinical diagnosis of knee OA per the American College of Rheumatology criteria and (iv) mild-moderate radiographic OA defined as Kellgren-Lawrence grade 2 or 3 on a postero-anterior fixed flexion knee radiograph taken using a positioning device (SynaFlexer; BioClinica) with medial compartment predominant disease [14–16]. Exclusion criteria were any history of previous lower limb fracture, previous knee surgery (including arthroscopy), history of inflammatory arthritis or contraindication to MRI or GBCA administration (e.g. pacemaker, renal failure). For HV participants, inclusion criteria were (i) age 40–60 years, (ii) no current or significant previous symptoms of knee pain or stiffness and (iii) BMI ≤ 35 kg/m^2^. At each study visit, participants completed the knee injury and osteoarthritis outcome score (KOOS) to assess symptoms and had their BMI recorded. No disease-modifying intervention was received by any participant during the study follow-up period.

### Image acquisition

Participants underwent MRI of a single knee (most symptomatic knee in OA participants, randomly selected knee in HV using a random number generator [www.random.org]) on a 3 T platform (GE 750; GE Healthcare) using an 8-channel transmit/receive knee coil (InVivo). Imaging was performed at baseline and 6-month follow-up. A subset of participants (10 OA, 6 HV) was asked to return for imaging at 1-month post baseline for assessment of test-retest repeatability. Participants were supine and their knee was positioned in the coil with padding and foot support to minimise subject motion.

All MRI sequence parameters are provided in Table 1 with further details in the Supplementary Materials.

**Table 1:** Selected MRI sequence parameters

Purpose	Sequence	TR/TE (ms)	FA (^o^)	Acquisition Matrix	FOV (mm)	Slice thickness (mm)	NEX
Synovial segmentation	3D SPGR FS	12/3.6	15	384 × 384^a^	320 × 320	2^c^	0.5
T_1_ mapping	3D SPGR	4/2.4	2/6/14	128 × 128^b^	320 × 320	4^d^	3
Dynamic series (35 phases)	3D SPGR	4/2.4	14	128 × 128^b^	320 × 320	4^d^	0.5
MOAKS assessment	Sagittal 2D IW FS FSE	1500/36	90	384 × 256	160 × 160	3 (1-mm gaps)	3
MOAKS assessment	Coronal 2D IW FS FSE	2500/36	90	384 × 256	160 × 160	3 ( 1-mm gaps)	3

*Abbreviations*: *MOAKS*, MRI osteoarthritis knee score; *SPGR*, spoiled gradient echo; *IW*, intermediate-weighted; *FS*, fat-saturated; *TR*, repetition time; *TE*, echo time; *FA*, flip angle; *FOV*, field-of-view; *NEX*, number of excitations

^a^Interpolated to 512 × 512 with zero filling

^b^Interpolated to 256 × 256 with zero filling

^c^Interpolated to 1 mm with zero filling

^d^Interpolated to 2 mm with zero filling

### Image analysis—pharmacokinetic modelling

Voxelwise pharmacokinetic modelling of DCE-MRI data was performed on registered images (Supplementary Materials) using the extended Tofts compartmental model [17] with a population-averaged arterial input function (AIF) [18]. All AIFs were corrected for individual patient haematocrit [19]. GBCA concentration was estimated from the change in signal relaxation due to the presence of GBCA (gadoterate [Dotarem]; Guerbet) compared to the native *T*_1_ values using a relaxivity of 3.5 L.mmol^-1^.s^-1^[20]. Native *T*_1_ values were calculated from the variable flip angle images acquired before the contrast agent injection [21]. The biomarkers extracted were *K*^trans^ (units min^-1^), the volume transfer constant for contrast agent between blood plasma and extravascular extracellular space; *v*_*p*_, fractional volume of blood plasma; *v*_*e*_, the fractional volume of extravascular extracellular space; and IAUC_60_ (mM.s), the initial area under the contrast agent concentration time curve 60-s post contrast agent arrival in the tissue.

### Image analysis—region of interest definition

**Fig. 1:**
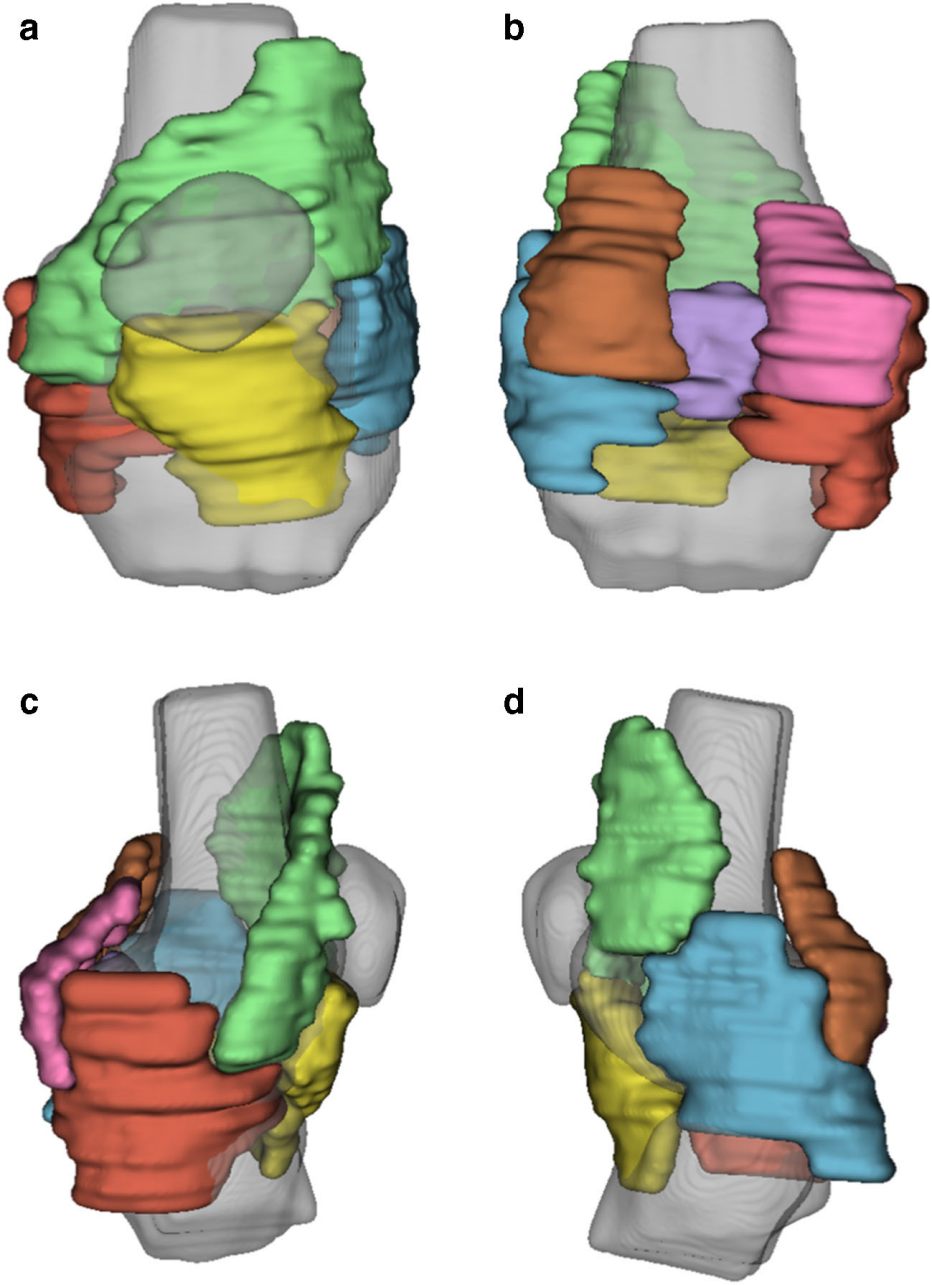
3D rendering of synovial regions of interest with anterior (**a**), posterior (**b**), medial (**c**) and lateral (**d**) views. 3D rendering of femur, tibia and patella (grey) provided for reference. ROI Key: green—suprapatellar, yellow—Hoffa fat pad, red—medial perimeniscal, blue—lateral perimeniscal, purple—intercondylar notch, pink—posterior medial femoral condyle, orange—posterior lateral femoral condyle

Two alternative methods of region of interest (ROI) definition were evaluated involving manual and semiautomatic approaches. Manual segmentation of the synovium was performed on the post-contrast 3D fat-suppressed spoiled gradient echo (FS SPGR) sequence by a musculoskeletal radiologist with 6 years’ experience (J.M.), with definition of seven synovial ROIs: suprapatellar, Hoffa’s fat pad, medial and lateral perimeniscal, intercondylar notch, medial and lateral posterior femoral condyles (Fig. 1). Anatomical definitions of synovial ROIs are provided in Table 2. The manual segmentation was intended to provide a rough estimation of where the synovium was located, rather than a detailed slice-by-slice manual segmentation.

**Table 2:** Anatomical definition of synovial regions of interest

Region	Definition
Suprapatellar	From mid pole of the patella to the superior extent of the suprapatellar bursa, including medial and lateral peripatellar recesses
Hoffa fat pad	From the junction of patellar tendon/tibial tuberosity to mid pole of patella superiorly. Posteriorly extends to the anterior aspect of intermeniscal ligament and anterior horns of medial/lateral menisci.
Medial perimeniscal	Wraps around medial meniscus. Extends superiorly deep to the medial collateral ligament (MCL) to the level of femoral MCL origin. Extends inferiorly deep to MCL to tibial MCL attachment.
Lateral perimeniscal	Wraps around lateral meniscus. Extends superiorly deep to lateral collateral ligament complex to fibular collateral ligament femoral origin. Extends inferiorly to level of tibiofibular joint. Includes popliteus recess, if present.
Intercondylar notch	From the superior aspect of anterior cruciate ligament (ACL) femoral origin to the floor of the intercondylar notch at intercondylar eminences. Posterior margin tangential to the most posterior aspect of posterior cruciate ligament, anterior margin at anterior aspect of ACL.
Posterior medial femoral condyle	From most proximal aspect of the medial head of gastrocnemius tendon to superior border of the posterior horn of the medial meniscus. Extends to meet intercondylar notch region laterally and medial perimeniscal region medially. Includes semimembranosus/medial head of gastrocnemius bursa, if present.
Posterior lateral femoral condyle	From most proximal aspect of the lateral head of gastrocnemius tendon to superior border of the posterior horn of the lateral meniscus. Extends to meet intercondylar notch region medially and lateral perimeniscal region laterally.

**Fig. 2:**
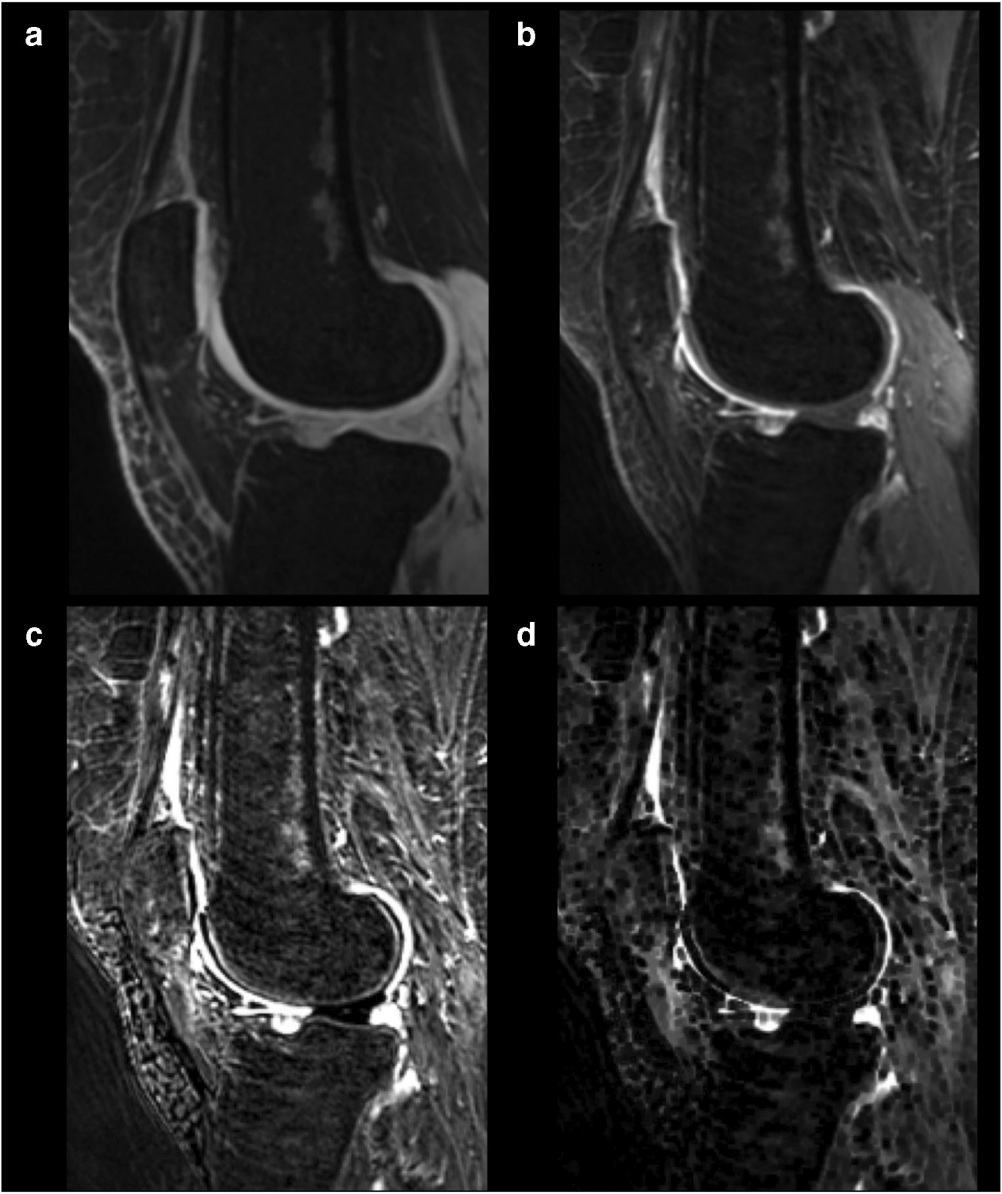
Example of the use of shuffle transform to improve quality of subtracted image compared to simple subtraction of registered images. **a** pre-contrast 3D FS SPGR, **b** post-contrast 3D FS SPGR, **c** simple subtraction (following intensity-based registration), **d** shuffle subtraction. Improved subtraction quality is seen when the shuffle transform is used

For semiautomatic segmentation, enhancing voxels were defined by subtracting the pre-contrast 3D FS SPGR sequence from the matching post-contrast sequence using a shuffle transform [22]. For a given voxel in the post-contrast image, the shuffle transform minimises the absolute difference between the signal intensity of that voxel and the corresponding voxel plus a defined neighbourhood (for this study the adjacent 3 × 3 voxels) in the pre-contrast image. This improves the quality of the subtracted images and is also robust to residual motion artefact following image registration (Fig. 2). The shuffle-subtracted images were then converted to binary enhancing masks using the Otsu thresholding [23]. The intersection between this binary mask and the manual segmentation was termed the ‘volume of enhancing pannus’ (VEP) mask. The VEP mask was used for the extraction of median DCE-MRI biomarker values for each synovial ROI and for the whole joint (all ROIs combined). In addition, the VEP mask was used to create an estimate of volume of synovial tissue (VEP, measured in mL) by multiplying the number of voxels included in the VEP mask by the voxel size.

Segmentation was repeated by the original observer with an interval of > 6 months between analyses and an independent second observer (T.R., a radiology resident with 4 years’ experience) for all baseline visits to enable assessment of intra and inter-observer reproducibility of DCE-MRI biomarkers.

### Image analysis—semiquantitative grading

Semiquantitative grading of synovitis was performed using the MRI Osteoarthritis Knee Score (MOAKS) by a musculoskeletal radiologist with 6 years’ experience (J.M.) [24]. MOAKS grades synovitis in two ways: signal alterations in Hoffa’s fat pad (Hoffa synovitis) and degree of suprapatellar joint effusion (effusion-synovitis). Both are scored on a 4-point ordinal scale (0–3). The intra and inter-reader reproducibility of MOAKS have previously been published [24].

### Statistics (see Supplementary Material for detail)

Test-retest repeatability was assessed using baseline and 1-month whole joint data with calculation of the intraclass correlation coefficient (ICC). Intra- and inter-observer reproducibility was assessed using the root-mean-square coefficient of variation (RMSCV) and the concordance correlation coefficient (CCC). We also calculated the smallest detectable difference (SDD), representing the magnitude of change that would give 95% confidence of a change being genuine rather than due to measurement noise, assuming identical measurement conditions. This is defined as 2.77 ($\sqrt{2}$ × 1.96) times the test-retest within-subject standard deviation or within-subject coefficient of variation (dependent on correlation between magnitude and variability of the biomarker) and is also known as the repeatability coefficient (RC) [25].

Discrimination between OA and HV participants was assessed using baseline data. Descriptive statistics were calculated for each group, and the standardised mean difference (SMD) was estimated for each DCE-MRI biomarker by dividing the difference in mean between the two groups by the pooled standard deviation.

Six-month changes in each biomarker were assessed using descriptive statistics. The number of participants with changes in each biomarker greater than the SDD was calculated.

No formal sample size calculation was performed for this feasibility study. All statistical analyses were performed in R version 3.6.1 [26].

## Results

### Participants

Fourteen OA and six HV participants were recruited. Baseline characteristics are provided in Table 3. Eight OA and six HV participants completed the 1-month visit. Twelve OA participants and five HV completed the 6-month visit. The reasons for the lost to follow-up were the inability to schedule the MR examination in the appropriate time window (*n* = 2) and participant withdrawal (*n* = 1).

**Table 3:** Participant characteristics at baseline. The Knee injury and Osteoarthritis Outcome Score (KOOS) is a validated patient-reported outcome measure which assesses knee pain and symptoms as well as the impact on knee-related activities of daily living, sports and recreation and quality of life. Scores range from 0 to 100, with 0 indicating the most severe symptoms possible and 100 indicating no symptoms

	Group	
	OA (*n* = 14)	HV (*n* = 6)
Age (years)^a^	51 (5)	55 (4)
Sex (M:F)	8:6	2:4
BMI (kg/m^2^)^a^	29.2 (4.1)	29.7 (3.0)
K-L grade (2:3)	10:4	N/A
KOOS pain^a^	60 (20)	98 (2)
KOOS symptoms^a^	56 (19)	96 (5)
KOOS ADL^a^	67 (21)	99 (1)
KOOS sports^a^	36 (23)	94 (7)
KOOS QoL^a^	33 (23)	95 (8)

*Abbreviations***:** *BMI*, body mass index; *K-L*, Kellgren-Lawrence; *ADL*, activities of daily living; *QoL*, quality of life

^a^Mean (SD)

### Test-retest repeatability

Repeatability metrics values for each parameter are provided in Table 4. Variabilities of *K*^trans^, IAUC_60_ and VEP were not significantly correlated with the value of the biomarker, so wSD and absolute SDD values are presented. Variabilities of *v*_*p*_ and *v*_e_ were significantly correlated with biomarker value, so wCV and percentage SDD values are presented. Kendall’s τ correlation coefficients for baseline and 1-month biomarker values are provided in Supplementary Table 1. Due to the poor repeatability of *v*_*p*_ and *v*_e_ and the presence of physiologically implausible values (e.g. *v*_e_ greater than 1), these biomarkers were not used for further analyses. Repeatability of biomarker measurements from semiautomatic segmentation (VEP mask) was better than those derived from manual segmentation for *K*^trans^ and IAUC_60_. Measurements derived from semiautomatic segmentation were therefore preferred for all subsequent analyses.

**Table 4:** Repeatability and reproducibility metrics for DCE-MRI biomarkers

		Repeatability (test-retest)						Reproducibility			
Parameter	Seg. method	${\sigma}_b^2$	${\sigma}_w^2$	ICC (95% CI)	wSD/wCV^a^	SDD	OA mean (SDD as %)	Intra-observer		Inter-observer	
								RMSCV (%)	CCC (95% CI)	RMSCV (%)	CCC (95% CI)
*K*^trans^ (min^-1^)	Manual	2.1 × 10^-5^	1.3 × 10^-5^	0.62 (0.14, 0.86)	0.004	0.010	0.020 (50)	3.0	0.99 (0.98,1.00)	3.0	0.99 (0.98,1.00)
	Semiauto	2.1 × 10^-4^	2.4 × 10^-5^	0.90 (0.71, 0.97)	0.005	0.013	0.039 (33)	2.1	1.00 (1.00,1.00)	2.1	1.00 (1.00,1.00)
IAUC_60_ (mM.s)	Manual	4.2	2.4	0.64 (0.18, 0.87)	1.54	4.26	4.71 (90)	3.4	0.99 (0.99,1.00)	3.2	1.00 (0.99,1.00)
	Semiauto	31.7	5.9	0.84 (0.58, 0.95)	2.43	6.74	9.47 (71)	2.4	1.00 (1.00,1.00)	2.4	1.00 (1.00,1.00)
Synovial volume^c^ (mL)	Manual	9845	820	0.92 (0.57, 0.98)	8.5%	23.5%	394 (NA)^b^	8.1	0.94 (0.85, 0.98)	16.1	0.74 (0.53, 0.87)
	Semiauto	474.5	667.0	0.40 (0, 0.75)	24.8	68.8	90.3 (76)	9.4	0.95 (0.88, 0.98)	9.1	0.94 (0.86, 0.97)
*v*_p_	Manual	4.1 × 10^-7^	7.4 × 10^-7^	0.36 (0, 0.69)	134%	371%	3.0 × 10^-4^ (NA)^b^	5.5	1.00 (1.00,1.00)	9.1	0.99 (0.99, 1.00)
	Semiauto	2.0 × 10^-6^	1.9 × 10^-6^	0.51 (0, 0.82)	152%	421%	5.4 (NA)^b^	11.7	1.00 (1.00,1.00)	6.0	1.00 (1.00,1.00)
v_e_	Manual	0	0.04	0 (0, 0.53)	65%	180%	0.36 (NA)^b^	7.0	0.97 (0.93, 0.98)	10.8	0.94 (0.88, 0.97)
	Semiauto	0.70	0.67	0.51 (0, 0.82)	97%	268%	0.63 (NA)^b^	6.8	1.00 (0.99, 1.00)	6.2	1.00 (0.99, 1.00)

*Abbreviations***:** *Seg*, segmentation; *ICC*, intraclass correlation coefficient; *wSD*, within-subject standard deviation; *wCV*, within-subject coefficient of variation; *SDD*, smallest detectable difference; *NA*, not applicable,${\sigma}_b^2$ between-subject variance; ${\sigma}_w^2$ within-subject variance; *RMSCV*, root mean square coefficient of variation; *CCC*, concordance correlation coefficient

^a^Provided as absolute values for wSD and percentages for wCV, with SDD correspondingly presented as an absolute value or percentage

^b^Median presented instead of mean as not normally distributed

^c^Synovial volume is synonymous with VEP for semiautomatic segmentation. Manual synovial volume includes both enhancing and non-enhancing voxels.

### Intra- and inter-observer reproducibility

Intra and inter-observer reproducibility was best for *K*^trans^ derived from semiautomatic segmentation (both RMSCV 2.1%, CCC [95% CI] 1.00 [1.00, 1.00]). *K*^trans^ and IAUC_60_ derived from semiautomatic segmentation demonstrated improved reproducibility compared to manual segmentation. All intra- and inter-observer reproducibility data are provided in Table 4.

### Discriminative ability

**Fig. 3:**
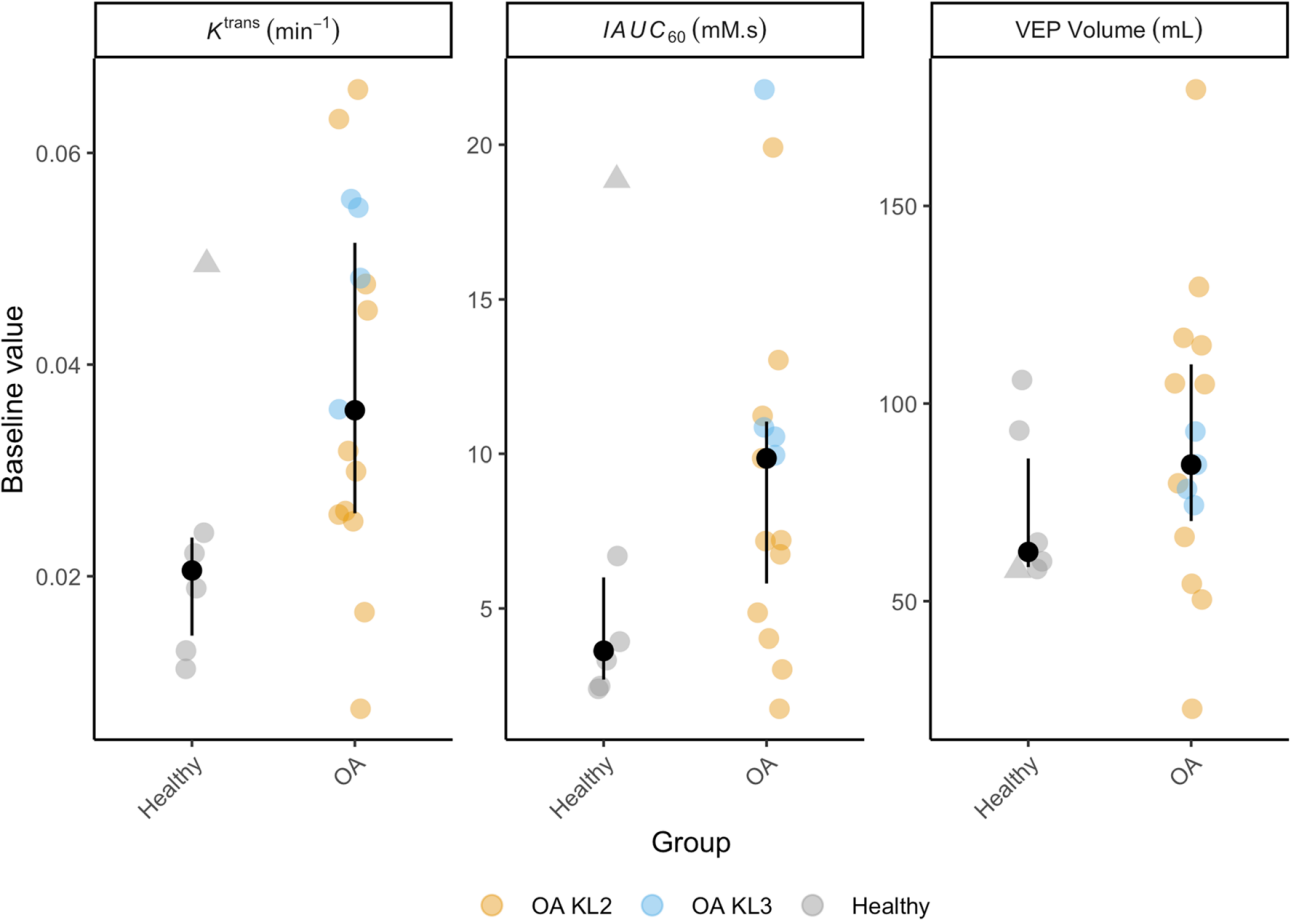
Baseline DCE-MRI biomarker values. Black dots are median values, with interquartile range error bars. The outlier HV is indicated with a triangle symbol (all other participants are circles)

Baseline between-group differences for the whole joint are illustrated in Fig. 3. Plots for individual ROI are provided in Supplementary Figure 1. One HV participant had much higher values of *K*^trans^ and IAUC_60_ than other HV participants (> 5 SD greater than mean HV value excluding this participant) across all ROIs. On further investigation, it was determined that this HV had taken part in karate practice the night before each of the three study visits and also had an undisclosed history of gout (never having affected the knee). Possible explanations considered for this value were that this represented part of the normal range of healthy values, or that the presence of gout or recent intense physical activity had confounded measurement. This participant’s data were not excluded because the participant met the pre-specified inclusion criteria, but, where appropriate, additional exploratory analyses excluding this participant’s data are reported.

SMDs between OA and HV groups were 0.94, 0.54 and 0.50 for *K*^trans^, IAUC_60_ and VEP respectively. Excluding the outlier HV case, SMDs were 1.34 for *K*^trans^ and 1.12 for IAUC_60_. Visual analysis of plots for individual synovial ROIs (Supplementary Figure 1) revealed the highest between-group differences for the intercondylar notch and medial and lateral perimeniscal ROIs for *K*^trans^ and IAUC_60_. The largest between-group difference and between-subject variability for VEP were seen in the suprapatellar ROI, as would be expected given the distensibility of the suprapatellar pouch to accommodate varying degrees of joint effusion. Discriminative ability was better in all cases for measurements derived from semiautomatic segmentation than for manual segmentation-derived measurements.

### Sensitivity to change over 6 months

**Fig. 4:**
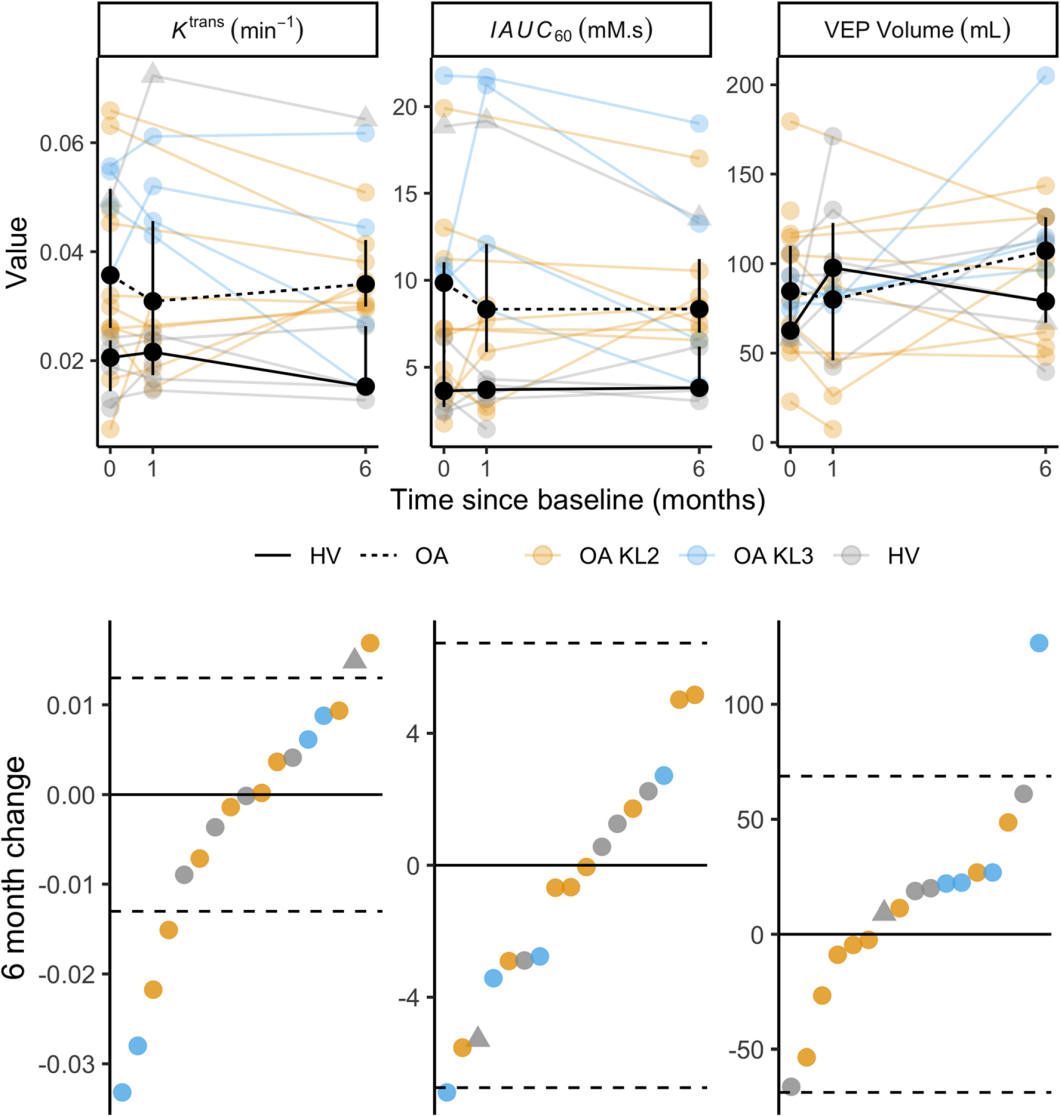
Top panel: Change in DCE-MRI biomarker values over time. Individual participant trajectories are displayed by partially transparent coloured circles and lines. Black circles and lines represent group median values (with IQR error bars). As for Fig. 3, the outlier HV is identified with a triangle symbol. Bottom panel: Waterfall plots of individual participant 6-month change in DCE-MRI biomarker values, ordered along the *x*-axis by magnitude of 6-month change value. Dotted lines represent +/-SDD

Changes in DCE-MRI biomarkers over 6 months are summarised in Fig. 4, with data for all synovial ROIs provided in Supplementary Figure 2.

**Fig. 5:**
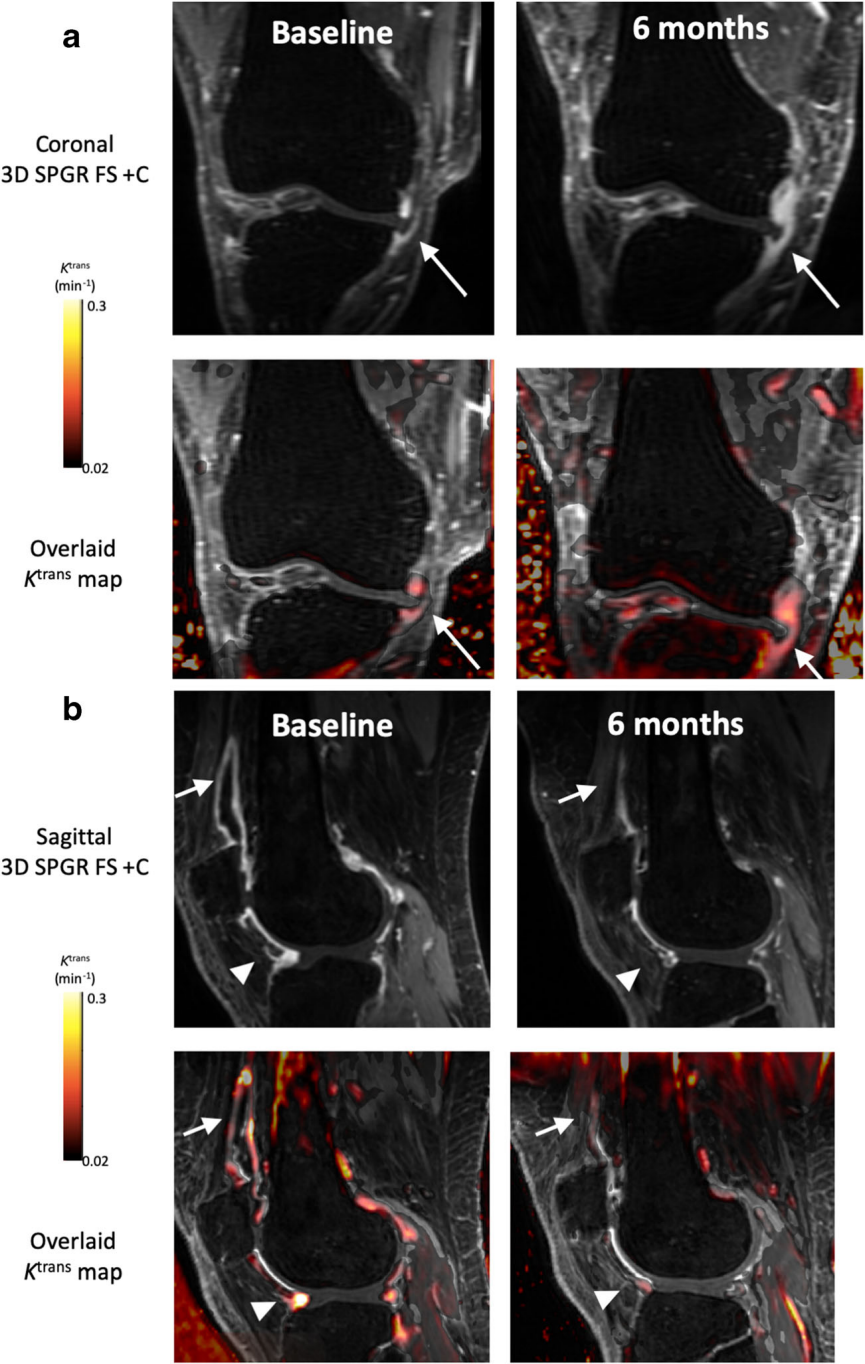
Example post-contrast 3D FS SPGR images overlaid with *K*^trans^ data from participants with increases (**a**) and decreases (**b**) in *K*^trans^ at 6 months which exceeds the SDD. In **a**, note extruded medial meniscus with cuff of adjacent synovitis (white arrow). At 6 months, the synovitis has increased both in amount and intensity. In **b**, note distention of suprapatellar pouch (white arrow) and synovitis adjacent to the anterior horn of lateral meniscus (white arrowhead) at baseline, with marked reduction at 6 months

For *K*^trans^, 5 out of 12 OA and 1 out of 5 HV participants had 6-month changes exceeding the SDD. For both IAUC_60_ and VEP, 1 out of 12 OA and no HV participants had changes exceeding the SDD. Using biomarkers extracted from manual segmentation rather than semiautomatic segmentation, 2 out of 12 OA participants and no HV participants had 6-month changes in *K*^trans^ exceeding the SDD, and no participants had 6-month changes in IAUC_60_ greater than the SDD. Representative images of participants with changes greater than the SDD are provided in Fig. 5.

**Fig. 6:**
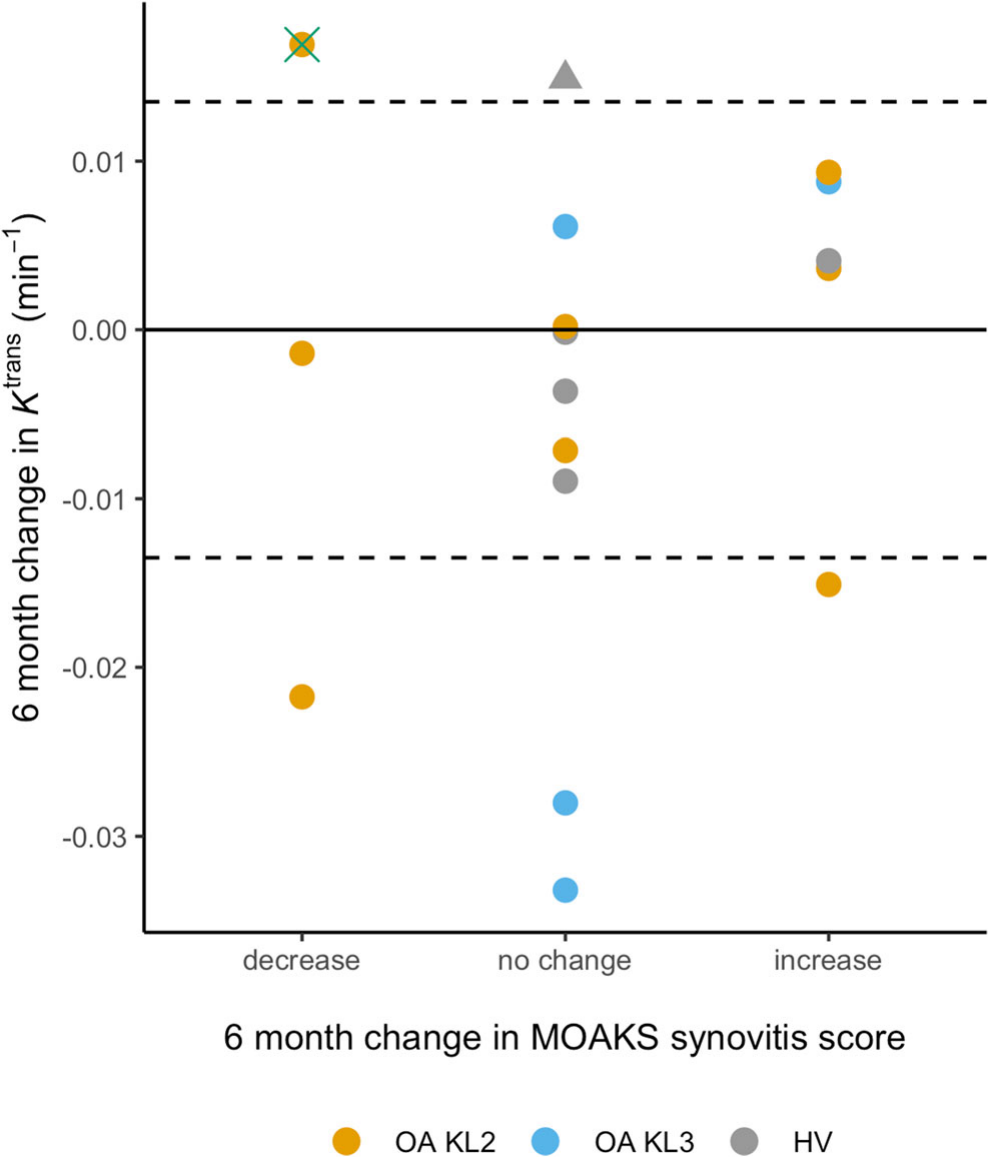
Individual participant 6-month changes in *K*^trans^ plotted against change in MOAKS synovitis score. Dotted lines are +/- SDD values for *K*^trans^. The green cross highlights the OA participant depicted in Fig. 5a who had discordant changes: an increase in *K*^trans^ exceeding the SDD, but a reduction in MOAKS synovitis score. The outlier HV is identified with a triangle symbol

A comparison of 6-month changes in *K*^trans^ and semiquantitative MOAKS synovitis score (sum of effusion-synovitis and Hoffa synovitis scores, scale 0–6) is provided in Fig. 6. There was limited concordance between participants with changes in *K*^trans^ exceeding the SDD and participants with changes in MOAKS synovitis score.

## Discussion

This study suggests that *K*^trans^ is the optimum of the evaluated DCE-MRI biomarkers for use in experimental medicine studies, with the best test-retest repeatability, best discrimination between OA and HV participants and greatest sensitivity to change as judged by the number of participants showing detectable changes over a 6-month period.

Several previous studies have used DCE-MRI to evaluate synovitis in knee OA, including describing cross-sectional associations with symptoms and longitudinal association with response to treatment [12, 27]. Novel contributions of the current work include (1) improved synovial segmentation leading to more precise parameter estimates, (2) assessment of test-retest repeatability which is required for the interpretation of change at an individual level, (3) assessment of inter-observer reproducibility and (4) comparison of DCE-MRI biomarker values between OA and healthy knees which is needed to assess discriminative validity and also to inform effect size estimations for interventional studies.

Biomarkers that assess the intensity of synovitis (*K*^trans^ and IAUC_60_) performed better than VEP, which reflects the extent of synovitis, across all assessment domains. This finding agrees with a previous knee OA study which suggested improved sensitivity to change of ‘intensive’ vs ‘extensive’ biomarkers of synovitis [12]. One possible explanation for the superiority of intensive biomarkers is the fact that synovial tissue may enhance despite not being actively inflamed, for example in areas of fibrosis related to previous inflammation [3]. The extensive biomarker can therefore be hypothesised to measure both active and inactive disease. However, such areas are likely to demonstrate different kinetic characteristics to areas of active inflammation, allowing intensive biomarkers to more accurately reflect disease activity at the time of the scan. DCE-MRI biomarkers derived from semiautomatic segmentation performed better than those derived from manual segmentation across the majority of assessment domains. Previous studies have demonstrated reduction in time taken for analysis with semiautomatic approaches but with similar repeatability and reproducibility to manual approaches [28, 29]. One plausible explanation for the demonstrated superiority of our semiautomatic approach is the fact that we used shuffle subtraction prior to our thresholding step, in contrast to approaches which attempt to threshold from the post-contrast images alone.

Interestingly, test-retest repeatability metrics for manual synovial segmentation were better than those for the semiautomatic approach. This probably relates to the fact that the manual segmentation was created to provide a rough mask of the location of the synovium which is then used by the semiautomatic method to identify enhancing voxels within the masked region. It is relatively straightforward for an expert radiologist to provide this initial rough mask as evidenced by the good intra and inter-observer reproducibility of manual segmentation. However, the manual method does not capture the variability in the volume of actual enhancing synovial tissue, in contrast to the semiautomatic method. The volume of enhancing synovial tissue (rather than the approximate region within which it is located) is more likely to undergo biological variation during the test-retest interval. Intra-observer reproducibility was similar for the two methods, but with superior inter-observer reproducibility for semiautomatic segmentation.

The design of our study assumes a natural history of OA with negligible change over one month (repeatability), but with the possibility of disease progression over 6 months. This is a short interval relative to the conventional concept of OA as a slowly progressive condition developing and progressing over years. However, experimental medicine studies are typically of short duration and so to be useful in this setting, an imaging biomarker has to be sensitive enough to detect changes over short intervals. We therefore chose a 6-month interval as a reasonable trade-off between the requirements of experimental medicine studies against the expected relatively slow change in disease.

There was a wide range of 6-month changes in DCE-MRI biomarkers in both positive and negative directions in OA participants. This may reflect the fluctuating nature of synovitis in OA, which is well recognised clinically [30]. Several participants demonstrated 6**-**month changes greater than the SDD (particularly for *K*^trans^) suggesting that sensitivity to change is adequate for experimental medicine studies performed over this interval. A possible counter-argument is that this sensitivity to change indicates that the background variability is too high to expect to be able to detect additive effects of therapy. Moreover, more participants demonstrated significant decreases rather than significant increases in *K*^trans^, likely related to regression to the mean. However, it should be noted that the majority of participants did not demonstrate significant reductions in DCE-MRI biomarkers and typically had higher values than age-matched controls suggesting that there is potential for improvement in these biomarkers with treatment. Moreover, the group mean 6-month changes in DCE-MRI biomarkers for OA participants was close to 0, after adjustment for baseline values (data not shown). This suggests that the effects of treatment may also be detectable at a group as well as at an individual level.

Our results suggest that DCE-MRI biomarkers are likely to be of use in experimental medicine studies featuring putative anti-inflammatory and immunomodulatory disease-modifying treatments. The data presented can be used to inform sample size calculation for further interventional studies. For example, using the observed standard deviation of 6-month change in *K*^trans^ in this study (~ 0.015 min^-1^), a group-averaged reduction of 50% of the difference between OA and HV mean values (~ 0.01 min^-1^) could be detected with 80% power and a type 1 error rate of 5% (one-sided) with a sample size of 24 participants per group, assuming an active treatment vs placebo repeated-measures study design. This is a clinically feasible reduction relative to a previous study of change in *K*^trans^ following intra-articular steroid administration [12].

Limitations of this study include the long test-retest interval (1 month) relative to the time over which clinical fluctuations in synovitis occur in OA. Therefore, the measured variability is likely to include contributions from both methodological and biological sources, and true methodological variability is likely to be lower. A second limitation is that the results presented are from a single centre and obtained with meticulous quality control; therefore, extrapolation to multi-centre studies should be done with caution. However, previous work suggests that DCE-MRI biomarkers can be used in such a setting with appropriate training, calibration and quality control [31]. In particular, the use of a semiautomated pipeline as described in this study for defining the synovial ROI is likely to improve robustness in the multi-centre setting compared with manual methods [32]. Finally, the number of included participants was low. While this was to some extent limited intentionally to mimic the conditions of an experimental medicine study, it does limit the precision of biomarker performance metric estimates. There is no ‘magic number’ of participants required for a repeatability study [25]. However, we would contend that the uncertainty in our repeatability estimates is low enough to allow them to be used for sample size calculation and interpretation of change at the individual level in future interventional studies.

In conclusion, this study has assessed the test-retest repeatability, discrimination between OA and ‘normal’ tissue characteristics and sensitivity to change of DCE-MRI biomarkers. *K*^trans^ demonstrates the best performance across these domains and is therefore the most likely to be useful in experimental medicine studies and other future therapeutic trials.

## Supplementary Information

ESM 1 (DOCX 2 mb)

## Abbreviations

DCE: Dynamic contrast-enhanced
GBCA: Gadolinium-based contrast agent
KOOS: Knee injury and osteoarthritis outcome score
OA: Osteoarthritis
SDD: Smallest detectable difference
SPGR: Spoiled gradient echo
VEP: Volume of enhancing pannus

## Declarations

### Guarantor

The scientific guarantor of this publication is Dr James MacKay.

### Statistics and biometry

One of the authors has significant statistical expertise.

### Study subjects or cohorts overlap

Some study subjects or cohorts have been previously reported in MacKay JW, Kaggie JD, Treece GM, et al Three-dimensional surface-based analysis of cartilage MRI data in knee osteoarthritis: validation and initial clinical application. *Journal of Magnetic Resonance Imaging*: 10.1002/jmri.27193. This manuscript reports on the same study cohort but focusses on analysis of articular cartilage in contrast to the current manuscript which describes a completely separate set of analyses of synovitis.

### Methodology

• prospective

• experimental

• performed at one institution
