# Supplementary material for: Dynamic contrast-enhanced MRI of synovitis in knee osteoarthritis: repeatability, discrimination and sensitivity to change in a prospective experimental study
Source: Eur Radiol. 2021 Feb 16;31(8):5746–58. doi: 10.1007/s00330-021-07698-z (PMC8270862; doi:10.1007/s00330-021-07698-z)

# Supplementary material

### Supplementary Methods

## Image acquisition

### Pre-contrast MRI sequences included variable flip angle T1 mapping using 3D spoiled gradient echo (SPGR) sequences and a high-resolution 3D fat-suppressed (FS) SPGR sequence. For the dynamic acquisition, a multi-phase 3D SPGR sequence was used with temporal resolution for the whole knee of 14 seconds. At the end of the 6th phase, gadoterate (Dotarem; Guerbet) was administered via a pump at a dose of 0.2 mmol/kg and a rate of 3 mL/s followed by a 50 mL saline chaser at the same rate. Imaging continued for a further 29 phases (35 phases total, acquisition time 8 minutes 10 seconds) following gadoterate administration. A post contrast 3D FS SPGR sequence with identical parameters to the pre contrast 3D FS SPGR was also obtained for synovial segmentation (further details below). The total acquisition time for DCE-MRI sequences was approximately 21 minutes. Sagittal and coronal 2D intermediate-weighted, fat-saturated fast spin echo sequences were also performed to aid semi-quantitative assessment of synovitis using the MRI Osteoarthritis Knee Score (MOAKS)[1].~~BLINDED~~

## Image analysis — image registration for pharmacokinetic modelling

Motion correction was performed for all images in each data set using the Advanced Normalization Tools software (http://stnava.github.io/ANTs). The post-contrast 3D FS SPGR images were used as the target. Each image was rigidly registered to the target image followed by a nonlinear registration using symmetric normalization. A mutual information similarity measure was used for both steps. The post-registration images were inspected and compared to the target image to ensure satisfactory motion correction.

## Statistics

Test-retest repeatability was assessed via a two-way random effects ANOVA of baseline and 1-month whole joint data with calculation of the intraclass correlation coefficient (ICC):

| $ICC=\frac{\sigma_{b}^{2}}{\left( \sigma_{b}^{2}+ \sigma_{w}^{2} \right)}$ | (1) |
| --- | --- |

where $\sigma_{b}^{2}$ is between-subject variance and$\sigma_{w}^{2}$ is within-subject variance. This is equivalent to ICC_2,1_ in Shrout & Fleiss notation[2].

Inter-observer reproducibility was assessed using the root-mean-square coefficient of variation (RMSCV) and the concordance correlation coefficient (CCC). The RMSCV is calculated as

| $RMSCV = \sqrt{\frac{\sum_{i=1}^{n} \left( \frac{\sigma_{i}}{\mu_{i}} \right)^{2}}{n}}$ | (2) |
| --- | --- |

Where $\sigma_{i}$ is the standard deviation of two measurements for subject *i*, and $\mu_{i}$ is the mean of the two measurements. The CCC is calculated as

| $CCC = \frac{\sigma_{1}\sigma_{2}\rho_{1,2}}{\sigma_{1}^{2}+\sigma_{2}^{2}+\left( \mu_{1}-\mu_{2} \right)^{2}}$ | (3) |
| --- | --- |

Where $\mu_{1}$ and $\mu_{2}$ and $\sigma_{1}^{2}$ and $\sigma_{2}^{2}$ and are the means and variances for each observer respectively, and $\rho_{1,2}$ is the correlation between the two observers’ measurements.

The distribution of each DCE-MRI biomarker at baseline was assessed visually via Q-Q plots and also via the Shapiro-Wilk test using p < 0.05 as the threshold for significant deviation from normality. Correlation between the difference of the baseline and 1-month measurements and the mean value was assessed visually and quantified using Kendall’s τ. If biomarker values were normally distributed and there was no significant correlation (p > 0.05), within subject standard deviations (wSD) are presented. If biomarker values were not normally distributed or if significant correlation was detected, within subject coefficients of variation (wCV) are presented. wCV is calculated using the within-subject variance of the log-transformed DCE-MRI biomarker values[3]. The wSD or wCV values were then used to calculate the smallest detectable difference (SDD), representing the magnitude of change that would give 95% confidence of a change being genuine rather than due to measurement noise, assuming identical measurement conditions. This is defined as 2.77 ($\sqrt{2}$ x 1.96) times the wSD or wCV respectively and is also known as the repeatability coefficient (RC)[3].

Discrimination between OA and HV participants was assessed using baseline data. Descriptive statistics were calculated for each group, and the standardised mean difference (SMD) was estimated for each DCE-MRI biomarker by dividing the difference in mean between the two groups by the pooled standard deviation:

| $SMD= \frac{\mu_{1}- \mu_{2}}{\sqrt{(\left( n_{1}-1 \right)\sigma_{1}^{2}+ \left( n_{2}-1 \right)\sigma_{2}^{2})/(n_{1}+ n_{2}-2)}}$ | (4) |
| --- | --- |

Where $\mu_{1}$ and $\mu_{2}$ and $\sigma_{1}^{2}$ and $\sigma_{2}^{2}$ and are the means and variances for each group, and *n_1_* and *n*_2_ are the number of participants in each group. Six-month changes in each biomarker were assessed using descriptive statistics. The number of participants with changes in each biomarker greater than the SDD was calculated.

### Supplementary References

1. Hunter DJ, Guermazi A, Lo GH, et al (2011) Evolution of semi-quantitative whole joint assessment of knee OA: MOAKS (MRI Osteoarthritis Knee Score). Osteoarthritis Cartilage 19:990–1002.

2. Shrout PE, Fleiss JL (1979) Intraclass correlations: uses in assessing rater reliability. Psychol Bull 86:420–428

3. Raunig DL, McShane LM, Pennello G, et al (2015) Quantitative imaging biomarkers: A review of statistical methods for technical performance assessment. Stat Methods Med Res 24:27–67.

4. R Core Team (2019) R: A Language and Environment for Statistical Computing. R Foundation for Statistical Computing, Vienna, Austria

### Supplementary Table 1

Correlation coefficients (Kendall’s τ) between baseline and 1 month repeat values for DCE-MRI biomarkers

| Parameter | Seg. method | Kendall’s τ | p value |
| --- | --- | --- | --- |
| *K*^trans^ (min^-1^) | Manual | 0.10 | 0.68 |
|  | Semiauto | 0.33 | 0.13 |
|  |  |  |  |
| IAUC_60_ (mM.s) | Manual | 0.41 | 0.06 |
|  | Semiauto | 0.13 | 0.59 |
|  |  |  |  |
| Synovial volume (mL) | Manual | 0.28 | 0.20 |
|  | Semiauto | -0.05 | 0.86 |
|  |  |  |  |
| *v*_p_ | Manual | 0.82 | < 0.001 |
|  | Semiauto | 0.79 | < 0.001 |
|  |  |  |  |
| v_e_ | Manual | 0.36 | 0.10 |
|  | Semiauto | 0.59 | 0.004 |

Abbreviations: Seg — segmentation

### Supplementary Figure 1

Baseline DCE-MRI biomarker values for individual ROIs. Black dots are median values, with interquartile range error bars. The outlier HV is indicated with a triangle symbol (all other participants are circles). **Abbreviations:** Hoffa — Hoffa’s fat pad, Med Men — medial perimeniscal, Lat Men — lateral perimeniscal, PMFC — posterior medial femoral condyle, PLFC — posterior lateral femoral condyle

1. *K*^trans^ (min^-1^)


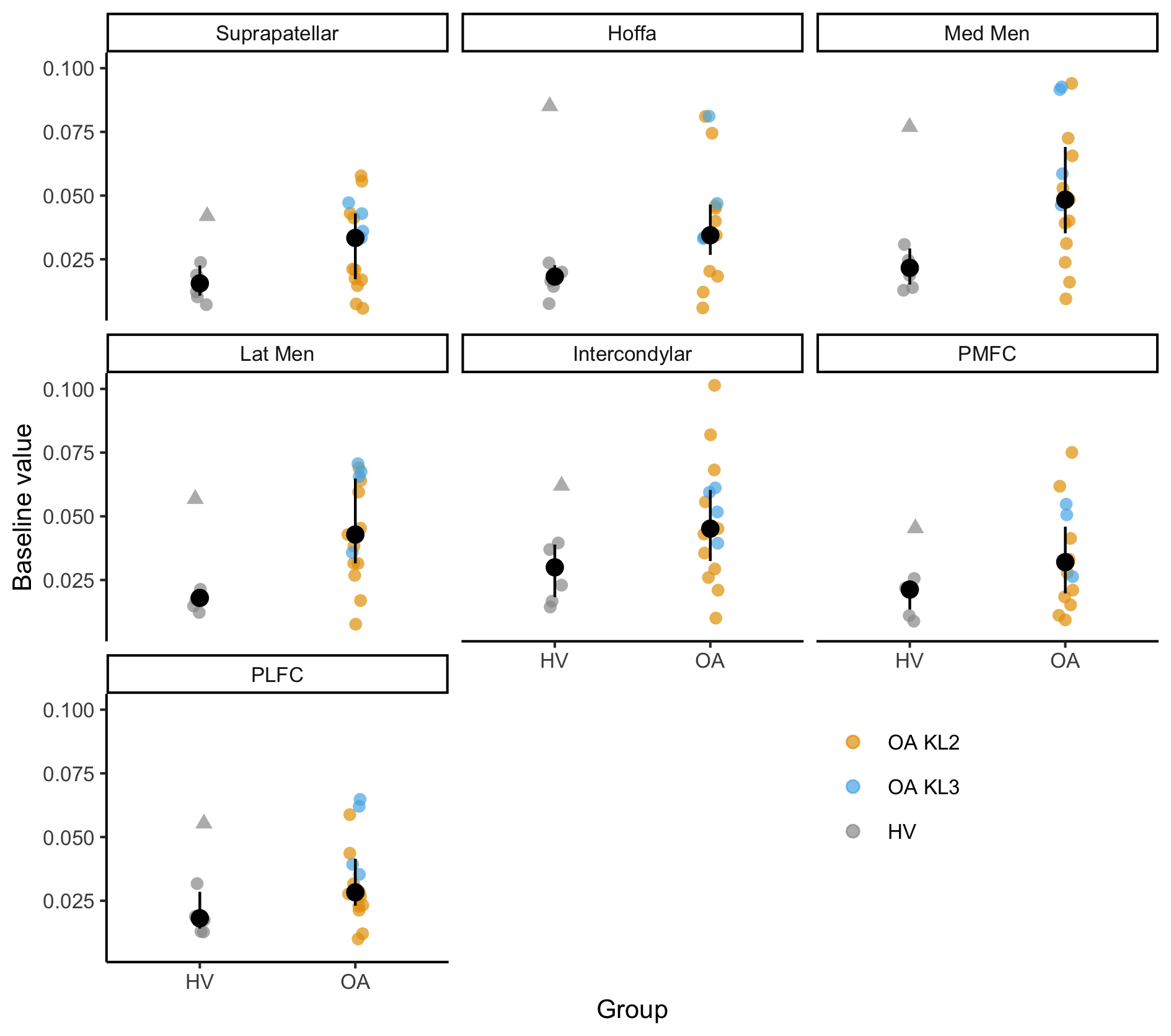


1. IAUC_60_ (mM.s)


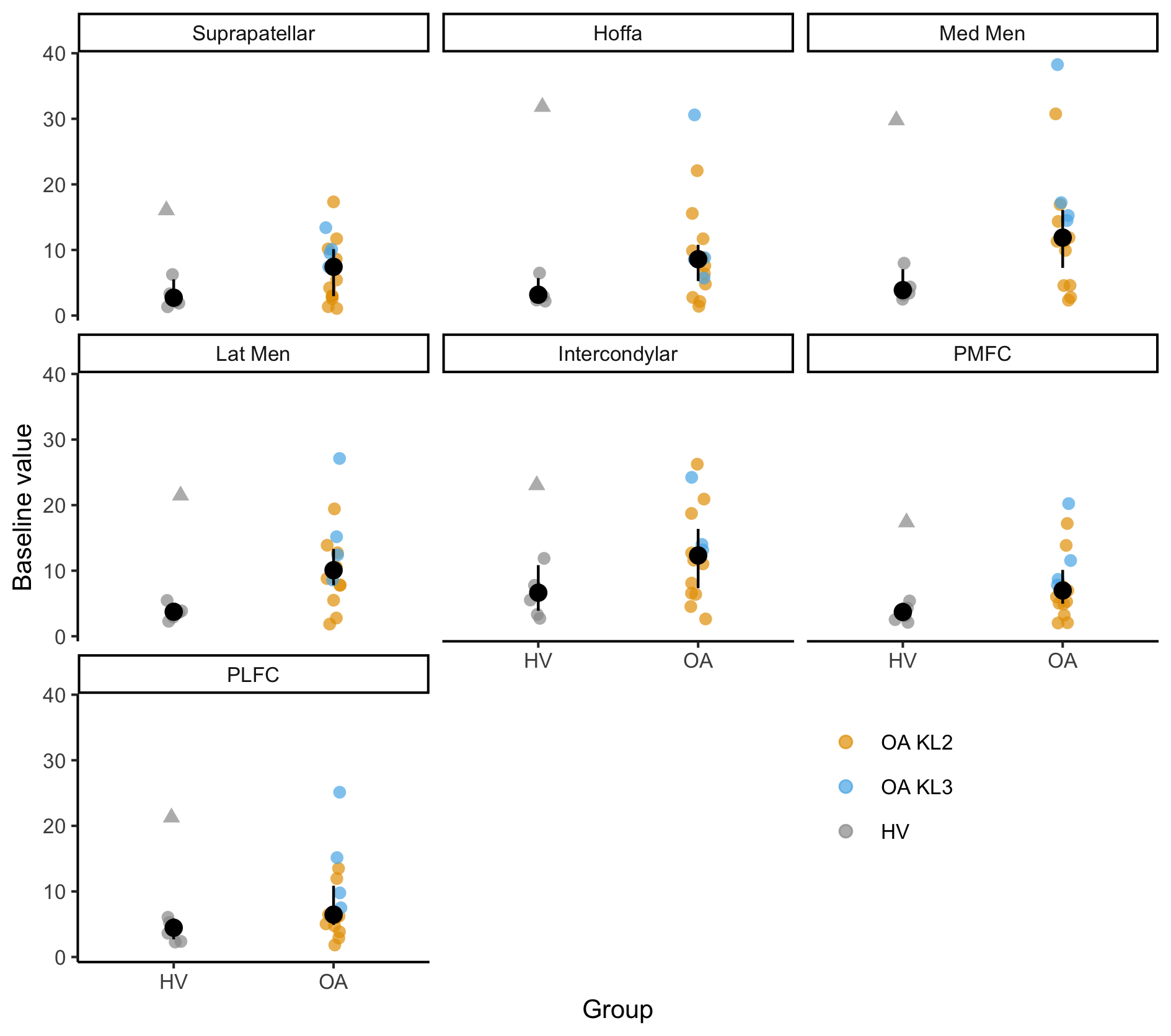


1. VEP (mL)


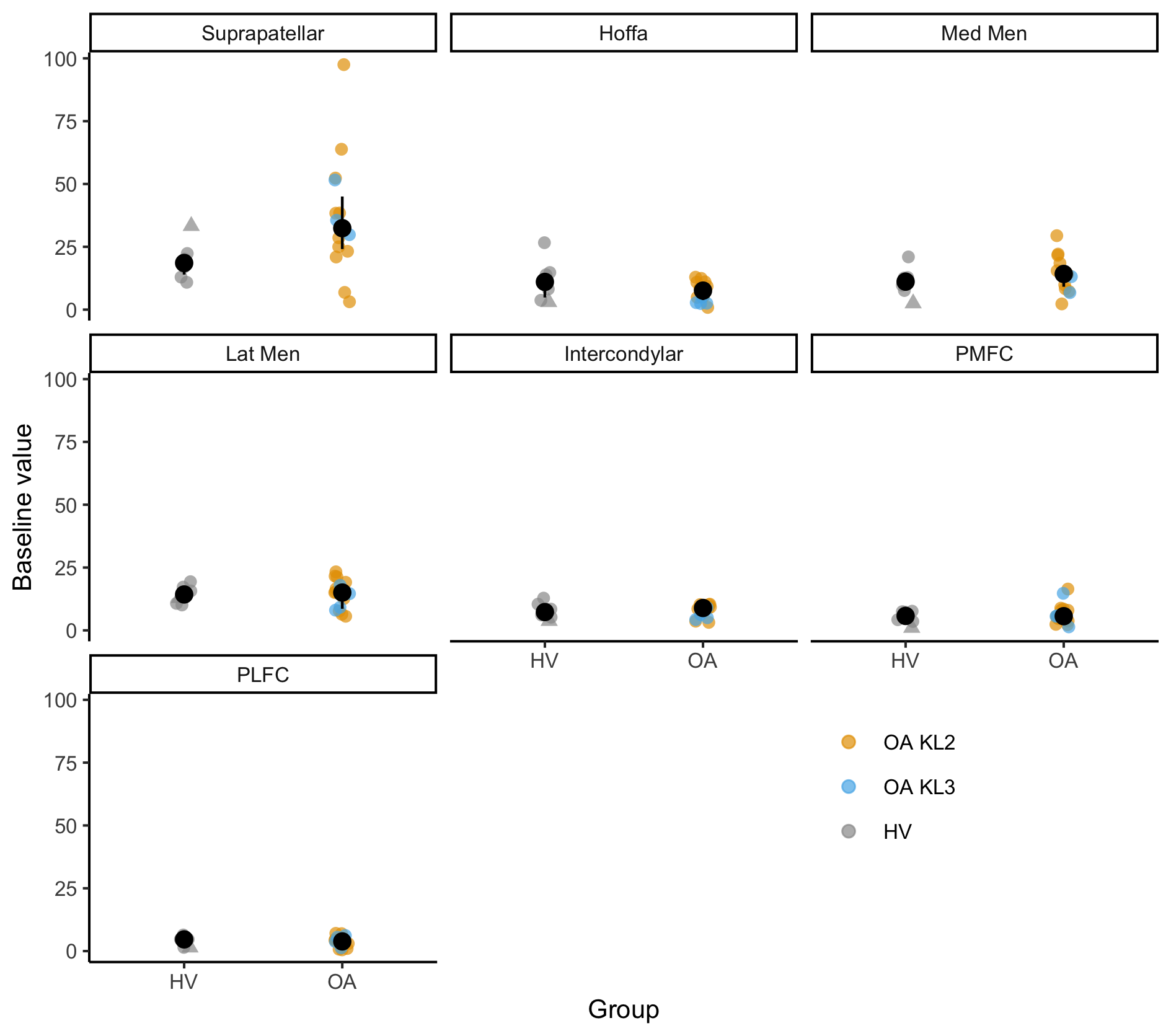


### Supplementary Figure 2

DCE-MRI biomarker values over time for individual ROIs. Individual participant trajectories are displayed by partially transparent coloured circles and lines, black circles and lines represent group median values (with IQR error bars). The outlier HV is identified with a triangle symbol. **Abbreviations** as for Supplementary Figure 1.

1. *K*^trans^ (min^-1^)


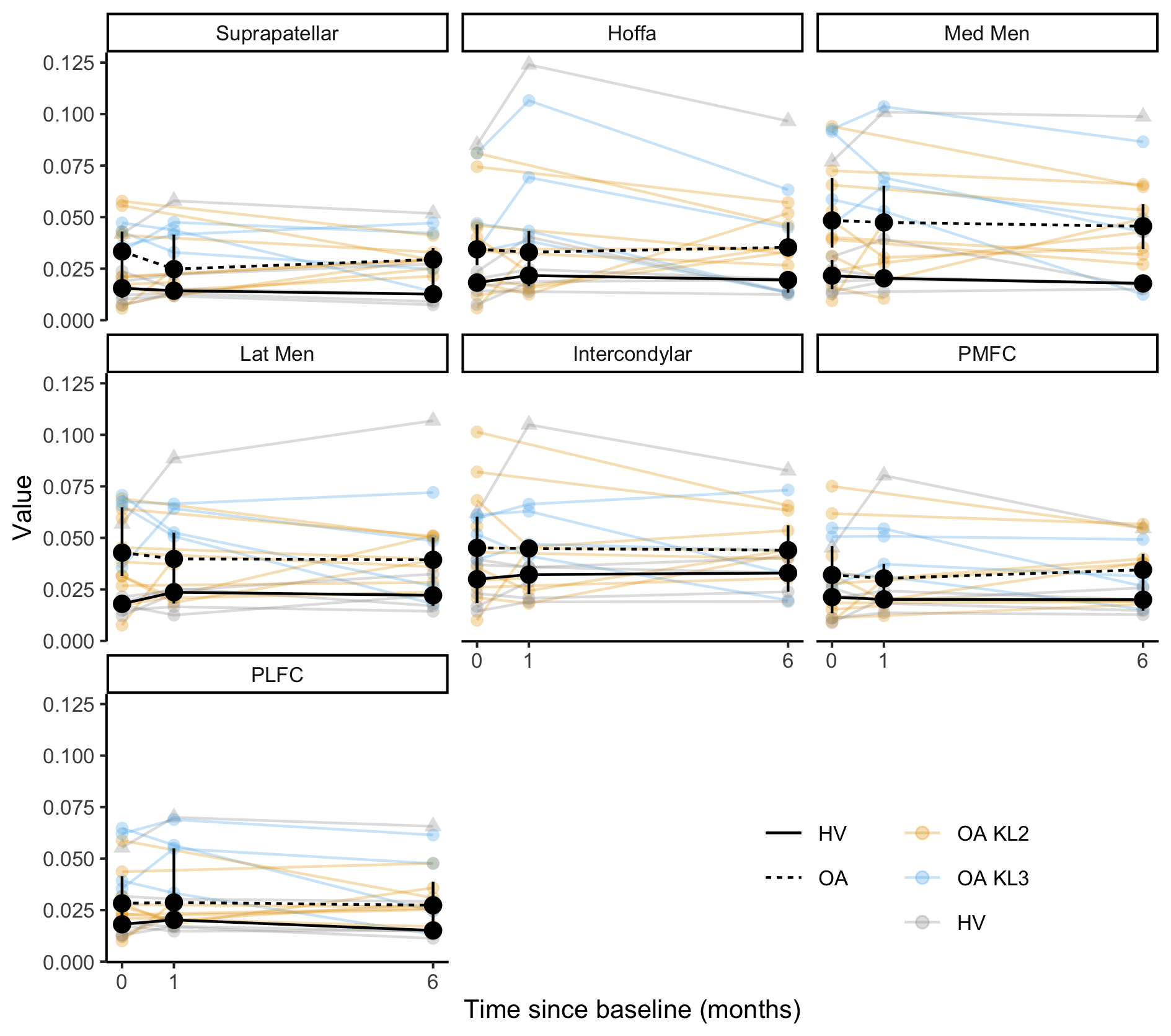


1. IAUC_60_ (mM.s)


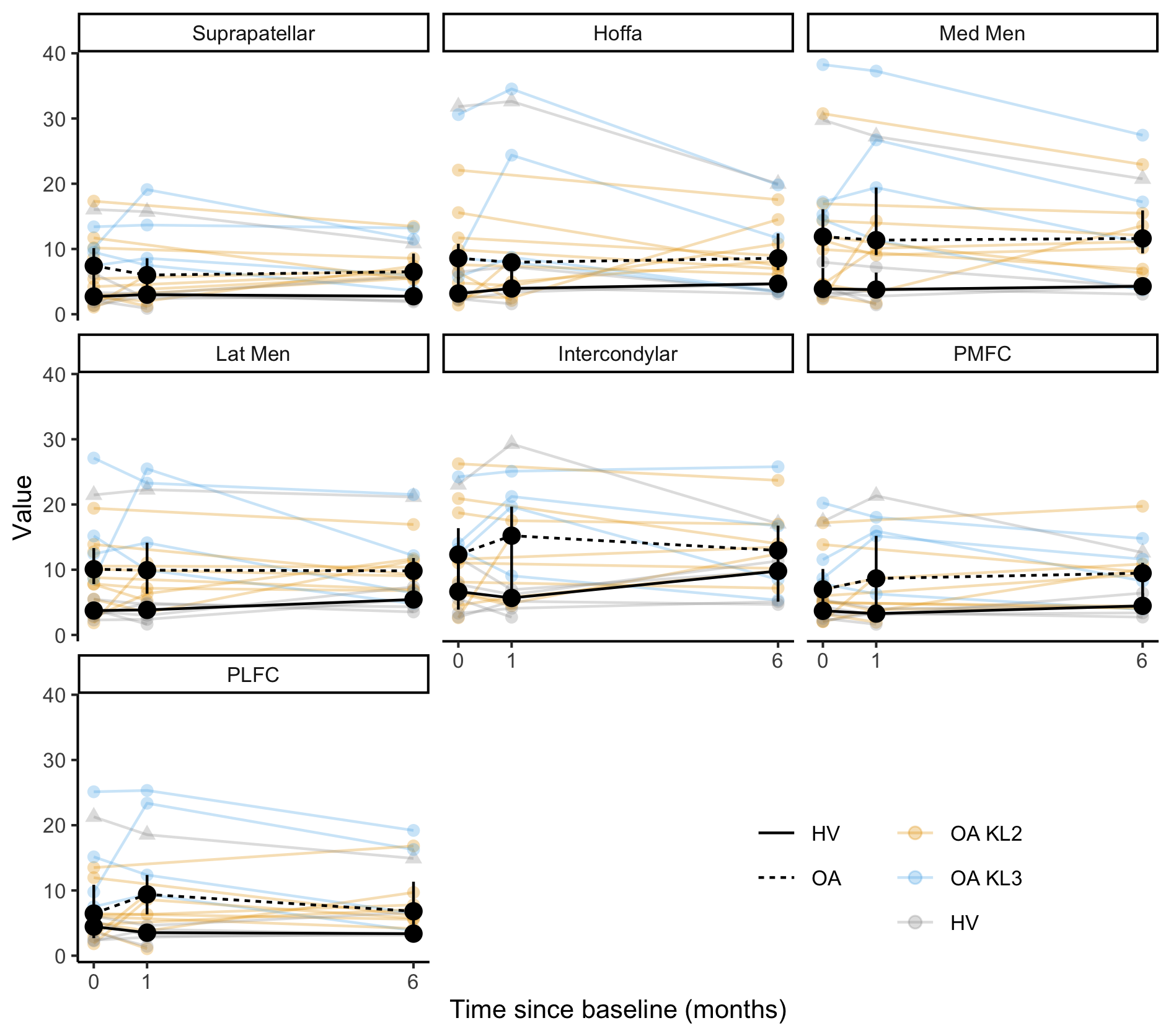


1. VEP (mL)


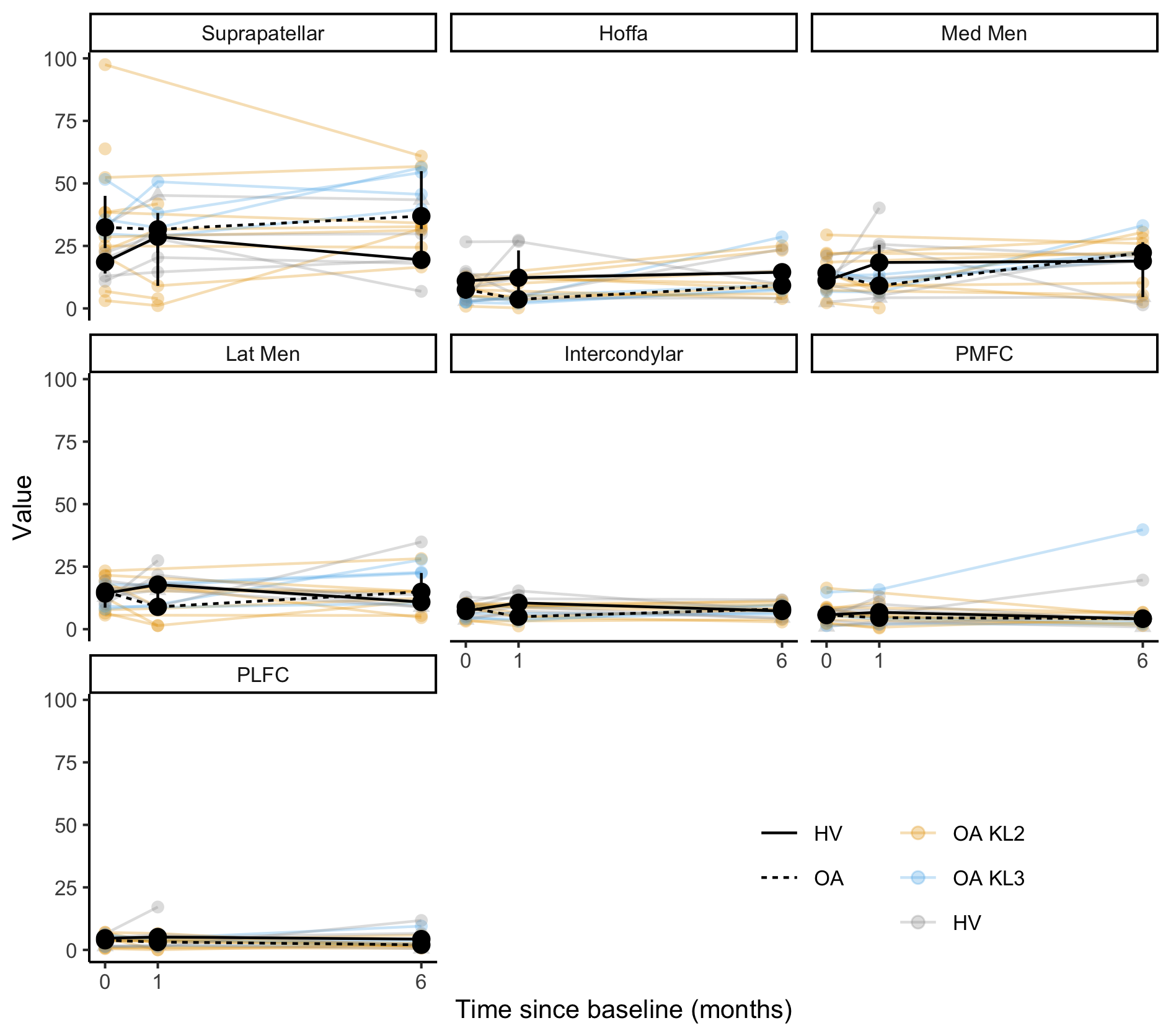

Supplement: Supplementary file 1 — (DOCX 2 mb) [file 330_2021_7698_MOESM1_ESM.docx]
